# Patient Participation and the Use of Ehealth Tools for Pharmacoviligance

**DOI:** 10.3389/fphar.2016.00090

**Published:** 2016-04-11

**Authors:** Joëlle Berrewaerts, Laure Delbecque, Pierre Orban, Martin Desseilles

**Affiliations:** ^1^Department of Psychology, University of Namur Medical SchoolNamur, Belgium; ^2^Department of Psychiatry, University of Montreal, MontrealQC, Canada

**Keywords:** pharmacovigilance, adverse drug reactions, adverse events, patient participation, web-based reporting, ehealth, intensive medication monitoring, mobile apps

## Abstract

In recent years, pharmacovigilance has undergone some major changes. First, the patient’s active role in identifying and describing adverse drug reactions (ADRs) has gained recognition. Second, pharmacovigilance has increasingly incorporated information and communications technology (ICT). Patients can now upload their own reports of ADRs online. Data on intensive medication monitoring are now collected via the Internet and smartphones. Worldwide collection of AEs using smart phones might become the leading technique in Low and Middle Income Countries where broad mobile phone service can be managed cheaper than Internet communication. At the same time, researchers are exploring the potential for data sharing via online forums and Internet search engines. In particular we synthetize the Pros and cons of the various methods for gathering pharmacovigilance data (i.e., Web-based spontaneous reporting of adverse drug reactions; Intensive drug monitoring studies; Analysis of online forum postings; Use of mobile phone systems to monitor drug effects). This article describes these advances and highlights their respective contributions.

## Introduction

Pharmacovigilance, or the practice of monitoring the effects of drugs, including the detection, assessment, understanding, and prevention of adverse events (AEs), is undergoing major changes. Until recently, pharmacovigilance drew mainly on spontaneous reports by health-care professionals (H), but lately, patients themselves are reporting adverse drug reactions (ADRs), and all these reports can be uploaded directly online via the Internet, tablets, and smartphones. Meanwhile, other ehealth tools are being used to complement these reports. Gathering as much information as possible on ADRs is vital for public health due to the significant consequences for health: ADRs account for 3% of hospitalisations in France, and they are one of the 10 leading causes of morbidity and mortality in the United States (USA) (Bousquet et al., 2011). The associated economic burden is also considerable. For example, in the USA, the cost exceeded $177.4 billion in 2000 alone (Ernst and Grizzle, 2001). However, most countries have not kept records of the cost of ADRs.

This article examines the contribution of patient participation and the use of ehealth tools for pharmacovigilance. The following questions are addressed:

– In what ways can patients participate in drug monitoring?
– Does patient participation improve pharmacovigilance?
– What ehealth tools effectively enable patient participation?
– Do ehealth tools contribute significantly to ADR detection?

A targeted literature search is presented, based on an article search within the MEDLINE database using the following keywords: pharmacovigilance AND ehealth (2)/pharmacovigilance AND Internet (72)/pharmacovigilance AND mobile phone (4)/pharmacovigilance AND smartphone (4)/pharmacovigilance AND apps (0), pharmacovigilance AND adverse drug reactions reported by consumers (18)/adverse drug reaction reporting AND patients AND Internet (38)/adverse drug reactions AND patient-reported (150)/drug monitoring AND mobile phone (62)/adverse drug reaction AND mobile phone (13).

The literature search covered articles published from January 2000 to January 2015. The MEDLINE search obtained 363 references. The abstracts of identified articles were then assessed for relevance. Studies were included if they contained theoretical or empirical data on patients’ reports of ADRs or investigations of ADRs using ehealth methods (e.g., Internet, smart devices, smartphones). Complete articles were obtained for each selected study. The references for each article were further searched to identify additional relevant articles. In all, 51 articles were included in the review.

## Patient Participation in Pharmacovigilance

Pharmacovigilance can be achieved by different methods, for example, cohort studies. However, the most commonly used method is spontaneous reporting of ADRs (Bandekar et al., 2010). This type of reporting has been described as an effective way to detect safety signals for medications (Ribeiro-Vaz et al., 2012). Most countries have a national pharmacovigilance program for gathering spontaneous ADR reports, analysing causal associations with drugs, and transmitting information to authorities. Currently, the Uppsala (2014) Monitoring Centre (the WHO centre for international drug monitoring, Uppsala, Sweden) is receiving data from120 participating countries. Data are pooled and ADRs are monitored using a data mining approach. Information on ADRs is then transmitted back to the national pharmacovigilance centers (Bandekar et al., 2010).

Until recently, European pharmacovigilance centers have relied exclusively on HCPs to report ADRs. However, since July 2012, the European Commission has allowed patients to make their own reports. It is notable that this decision lagged far behind those of other countries, including Australia, Canada, New Zealand, and the USA, which have been taking patients’ reports into account since the 1960s (van Hunsel et al., 2012).

The new European regulations reflect the fact that in pharmacovigilance, as in other healthcare areas, growing importance has been given to patient-reported information. According to Banerjee and Ingate (2012), patient-reported outcomes (PROs) via Internet channels have become increasingly important, enabling regulators to understand the real-world benefits and risks of medications. Nevertheless, despite the greater attention that patient reports are receiving, their full potential has yet to be realized in pharmacovigilance (Banerjee et al., 2013). At this point, safety reports and risk assessment procedures depend heavily on HCPs, despite the known limitations. For instance, underreporting by HCPs is a well-known problem (Hasford et al., 2002; Hazell and Shakir, 2006; Lopez-Gonzalez et al., 2009; Gonzalez-Rubio et al., 2011; Hanafi et al., 2012). Yet, given the clinical relevance of real-world data, patient reports could make a significant contribution to advance the knowledge on drug benefits and risks. Against this background, the Patient-Reported Outcomes Safety Event Reporting (PROSPER) Consortium was convened. Its aim is to improve safety reporting by better incorporating the patient’s perspective. PROSPER includes representatives from industry, regulatory authorities, the private sector, and academia as well as patients. It supports wider use of patient-reported outcomes of adverse events (PRO-AEs), and it has recently (from July 2011 to March 2013) developed guidelines, including the benefits of wider use and approaches for data capture and analysis.

In sum, the vital role that patients can play in the identification, description, and ultimately prevention of drug-related harm is increasingly being valued.

## Use of Ehealth Tools to Facilitate Patient Participation in Pharmacovigilance

Another recent development is the use of information and communication technology (ICT) tools to gather data on the AEs of marketed drugs. According to Cobert and Silvey (1999), Internet-based methods to ensure drug safety and pharmacovigilance are spreading rapidly. Pharmacovigilance agency websites are ever more welcoming of spontaneous ADR reports. Meanwhile, new mobile apps are being developed to allow ADR reporting anywhere, anytime. Besides spontaneous reports, studies have proposed more intensive ADR monitoring by following specific patient groups over time, which would also involve web-based data gathering. In addition, some researchers (Schröder et al., 2007; White et al., 2013; Abou Taam et al., 2014) have advocated online forum analysis as a supplementary ADR data source.

The remainder of this article describes these new approaches.

### Spontaneous Online Reports of ADRs

Inch et al. (2012) noted that national pharmacovigilance programs are welcoming more patient reports of ADRs. Moreover, these reports are more commonly submitted online in countries such as the USA, Canada, Australia, New Zealand, Kenya, and Malaysia. The US Food and Drug Administration (FDA) has been gathering ADR reports from both HCPs and consumers since its inception in the 1960s. Reports can be submitted by phone or email or uploaded directly online.

The ability to report online has increased the number of reports, especially by HCPs. For instance, Abadie et al. (2014) noted that once online notifications were introduced, reports by ambulatory HCPs increased by 45%.

In Europe, ways for HCPs to report ADRs online are proliferating. For example, in Belgium, the Federal Agency for Medicines and Health Products (FAMHP) has provided ADR report forms on its website since 2012. Since 2008, Swedish consumers can report ADRs to the Medical Products Agency (MPA), and since 2002, reports can be sent via an online form to the independent non-profit organization called the Consumer Association for Medicines and Health (KILEN). The United Kingdom (UK) has set up a reporting site called the Yellow Card Scheme (YCS) (Avery et al., 2011), where reports are passed on to the Medicines and Healthcare products Regulatory Agency (MHRA) by mail, phone, or Internet. To further develop pharmacovigilance capabilities, Europe has initiated an online platform called European Union Adverse Drug Reactions (EU-DR). The aim is to facilitate data access, data management, and data search to enable more in-depth examinations of ADR risks (Oliveira et al., 2013). The Netherlands, Denmark, Italy, and France have also developed online reporting systems (Herxheimer et al., 2010). However, some websites do not provide an online reporting form as such: visitors must download a form, complete it, and e-mail it.

So far, each country has been using its own ADR reporting system, and they do not all have the same data. Bandekar et al. (2010) compared online reporting forms across 10 different countries (India, Pakistan, USA, Great Britain, Kenya, Malaysia, Canada, Australia, South Africa, and Tanzania) in terms of 18 points deemed essential for good ADR reporting (e.g., information on allergies, diagnosis, drug dose and frequency, adequate space provided to capture reactions). Of the analyzed forms, Malaysia earned the highest score (16 out of 18 points). Unlike most of the other countries, its form addressed severity and causality. At the other extreme, Pakistan earned only 6 out of 18 points. This study reveals the need to harmonize ADR reporting forms across countries.

The above-cited studies attest that Europe and many other countries are gradually improving their online systems for patient reporting of ADRs.

Once it has been decided to involve patients in the pharmacovigilance process, a number of questions arise:

– Compared to reports by healthcare professionals (HCPs), do patient reports make a significant contribution to pharmacovigilance?
– What motivates patients to make spontaneous reports?

#### The Contribution of Spontaneous Patient Reports

Overall, studies that considered paper, online, and phone reports have shown that patient reports provide useful information that supplements HCP reports and helps identify new potential ADRs (Blenkinsopp et al., 2007; van Geffen et al., 2007; van Hunsel et al., 2009; Avery et al., 2011; Vilhelmsson et al., 2011).

Inch et al. (2012), in a systematic review of comparative studies of patient and HCP reports (paper, online, and phone) to national pharmacovigilance schemes, showed both similarities, and differences across schemes. Patients and HCPs reported similar percentages of serious ADRs in total numbers of ADR reports, but this percentage differed across studies. The Netherlands and the UK showed similarities in terms of reported drugs. The UK and Denmark showed clear differences between patients and HCPs in the body systems affected by ADRs, despite the considerably similar nature of the ADRs. The Dutch study also showed that patients and physicians reported ADRs for similar types of drugs.

Avery et al. (2011) noted that previous studies showed differences between patient and HCP reports in terms of reported drug types and reactions. They compared reports (paper, online, and phone) submitted by 5,180 patients and 20,949 HCPs from October 2005 to September 2007 to the YCS. Compared to HCP reports, patient reports contained a higher median number of suspected ADRs per report, with more detailed descriptions. Proportions of “serious” reports were similar for patients and HCPs, but drug and reaction patterns differed. Patient reports contained more detailed descriptions of reactions and more mentions of the effects on patients’ lives. Combining patient and HCP reports would generate new potential signals compared to HCP reports alone. Supplemental reports by patients have enabled identifying 47 new serious reactions not previously included in “Summaries of Product Characteristics”.

Some studies have examined reports on specific medication types. They also found differences between patient and HCP reports, highlighting the contribution of patient-provided information. For example, van Geffen et al. (2007) focused on ADRs associated with antidepressants in a comparison of patient- and HCP-reported ADRs via an Internet-based system of reporting to the Netherlands Pharmacovigilance Centre Lareb from May 2004 to May 2005. Compared to HCP reports, patient reports more often mentioned apathy, excessive sweating, ineffectiveness, somnolence, insomnia, sexual problems, and weight gain. In conclusion, patient and HCP reports may differ in the nature of ADRs. Vilhelmsson et al. (2011, 2012) also examined reports on antidepressants. Vilhelmsson et al. (2011) analyzed all reports submitted from January 2002 to April 2003 to an open Swedish website where anyone could report their experience with medications. Of a total of 665 reports, 442 concerned antidepressants, representing 2,394 ADRs, of which 878 (37%) were psychiatric effects. The conclusion was that consumer reports may contribute important information on more serious psychiatric ADRs following traditional antidepressant treatment. In another study, Vilhelmsson et al. (2012) interpreted the content of 181 online reports submitted to KILEN from January 2002 to April 2009 and concerning suspected ADRs for antidepressants. The results showed that free text comments in patient report forms can be valuable for pharmacovigilance, and can provide important information on how drugs may affect individuals and their lives. The majority of patient reports mentioned symptoms of mental disturbances (sometimes severe), which affected them in various ways, especially when the medications were discontinued. Patients often stated that they received no information on potential ADRs from their doctor, and what is more, received no follow-up. Some patients also reported losing confidence in their doctors, who did not believe their reports of experienced ADRs, which led patients to attempt to discontinue treatment.

van Hunsel et al. (2009) analyzed ADR reports on statins, a class of drugs used to help lower blood cholesterol levels. They examined reports submitted to the Lareb Centre in the Netherlands after a TV program called *Radar* was broadcast on the benefits and risks of statins. The authors compared online patient reports with online and paper reports by HCPs. Results suggested that the media attention temporarily increased patient reports, whereas HCP reports remained as usual. In terms of seriousness and number of drug cessations, patient and HCP reports were similar. However, patients reported non-recovery more often than HCPs.

In light of these studies, consumer and HCP reports appear to differ both quantitatively and qualitatively. At the same time, the supplementary information provided by patients was considered useful for improving drug monitoring.

Given the attention that is being paid to patient reports, it would be important to better inform consumers about reporting systems. At this point, few patients know that they can submit self-reports of ADRs. For example, in the UK, the majority of patients are unaware of this possibility (Fortnum et al., 2012 cited in van Hunsel et al., 2012). In 2009, only 8.5% of individuals knew about the YCS (Avery et al., 2011). In Korea, Lee et al. (2012) analyzed spontaneous online reports from January to December 2008. Of the 933 cases of ADRs reported, 53% were made by doctors, 31% by pharmacists, 13% by nurses, and only 3% by the public. In addition, the number of patient reports varies widely across countries. For example, a considerably higher percentage of patients submits reports (mail, phone, and online) in Denmark. In 2008, of 2,925 reports received, 72% were made by doctors, 2% by pharmacists, 6% by other HCPs (nurses, dentists), and 19% by patients (Herxheimer et al., 2010). van Hunsel et al. (2012) noted that most of the 11 countries included in their review (Australia, Canada, Denmark, Malaysia, the Netherlands, New Zealand, Norway, the Philippines, Sweden, the UK, and the USA) ideally wanted to spend more on public awareness campaigns to generate more patient ADR reports.

#### Patient Motivations to Make Spontaneous Reports

For purposes of increasing consumer participation, it would be useful to gain a deeper understanding of what motivates patients to make spontaneous reports. In this perspective, van Hunsel et al. (2010) investigated the motives behind patient reports of ADRs to a pharmacovigilance center in the Netherlands. An online questionnaire was sent to 1,370 patients who had made previous reports from January 2008 to March 2009, for a response rate of 76.5%. The main motives for patient reporting were a desire to share their experience, the severity of the ADR, worries about their own situation, and the lack of warning information in the patient leaflet. Of the respondents, 93.8% agreed that ADR reports can prevent harm to other people, 97.9% believed that their reports contributed to research and knowledge, 90.7% felt responsible for reporting ADRs, and 92.5% intended to report ADRs in future. These concerns are similar to those observed in HCPs. Biriell and Edwards (1997) explored positive reasons for physicians and pharmacists to take the time to report ADRs, both online and not. The reasons were classified into 14 categories, with most falling into the top six: motivation to contribute to medical knowledge, reaction previously unknown to the reporter, reaction to a new drug, all significant reactions reported, known association between a drug and a reaction, and severity of the reaction.

Aside from motivations, it would be instructive to understand more about the factors that influence consumers to report ADRs. These factors could be somewhat similar to those that influence HCTs. For example, Lopez-Gonzalez et al. (2009) conducted a systematic review of the underlying determinants for HCPs. They found that medical speciality was the professional characteristic that was most closely associated with under-reporting in 76% of studies involving physicians. Other factors associated with under-reporting were the fact that only the most severe ADRs were reported, diffidence (fear of ridicule for reporting merely suspected ADRs), lethargy (a combination of procrastination, lack of interest or time to find a report card, etc.), indifference (it is almost impossible to determine whether a medication is responsible for a particular ADR), and complacency (only “safe” drugs are assumed to be allowed on the market).

It appears that patients are driven by many motivations to make spontaneous ADR reports. Some patient motivations are similar to those expressed by physicians, such as severity of the ADR, a desire to contribute to medical knowledge, and the fact the certain ADRs are not yet well known or mentioned in the explanatory leaflet. However, patients also appear to have purely altruistic motives.

Apart from spontaneous ADR reports, more systematic and targeted data gathering methods have been implemented to monitor ADRs for specific marketed drugs. Generally known as intensive medication monitoring, these methods complement spontaneous reports. They involve observing a cohort over time without intervention in order to monitor a particular drug. The advantage is that it allows gathering follow-up data over time, which is rarely the case for spontaneous reports. Intensive monitoring systems have been set up in the Netherlands, the UK, and New Zealand, for example.

### Web-Based Intensive Monitoring

Härmark and van Grootheest (2012) presented an overview of the results of web-based intensive monitoring as well as the pros and cons. This method uses a specific inclusion point, such as an eligibility criterion that patients are using a medication for the first time, and eligible patients are identified when they present their prescriptions at pharmacies. Patients are followed over time using web-based questionnaires to gather information about drug use and ADRs. The main expert opinion is that this type of intensive monitoring, which lets patients be the information source, can be useful for postmarketing monitoring. For instance, it can address other aspects besides ADRs, such as indications for use and off-label use, dosage, and compliance.

According to Härmark et al. (2011a), web-based intensive monitoring allows gathering longitudinal safety data on drugs and examining the time course of ADRs. It can make a valuable contribution to pharmacovigilance because it generates more types of information compared to spontaneous reports and other intensive monitoring methods. For example, in the case of pregabalin, Härmark et al. (2011b) showed that a web-based intensive monitoring system can advance the knowledge on ADRs, such as headache, while providing quantified data and information on the time to onset of the ADR and its evolution over time. Participants were first-time users of pregabalin and were identified by their prescription filled at participating pharmacies. After online registration, participants received emailed questionnaires 2 weeks, 6 weeks, 3 months, and 6 months after beginning treatment. This type of system can also be used to identify signals that merit further investigation, for example, abdominal pain or potential interactions with oral antidiabetics. In another study, Härmark et al. (2011c) used a similar web-based intensive monitoring system in over 3,000 patients who were vaccinated against the pandemic influenza A (H1N1) virus in general practice. They found that more than a third of the patients (37%) reported adverse events following immunisations (AEFIs). No unexpected serious reactions were reported, nor were signals of possible new AEFIs. Broos et al. (2010) also demonstrated the usefulness of an online survey for rapid gathering of information about vaccine safety, in this case, the H1N1 vaccine administered to children aged 6 months to 4 years. From a survey emailed to the vaccinated children’s parents who reported fever to Lareb after the first immunization, the researchers found that 56.2% of these children had a fever that peaked at from 39.0 to 40.0 40°C, of which 70.4% recovered within 2 days.

According to Härmark et al. (2013a), little is known about patients’ motivations to participate in web-based postmarketing monitoring. They analyzed the motivations of patients who participated in a web-based intensive monitoring study on the safety of antidiabetic drugs (excluding insulins). In all, 1,332 patients responded to an online questionnaire. The main motive for participating was altruism, whereas experiencing ADRs or negative experiences with drugs were generally less important. The type of patient also played a role: compared to women, men felt that the potential future benefits were more important. The overall opinion about the system was positive. In another study, Härmark et al. (2013b) compared diabetic patients who participated in web-based intensive monitoring [i.e., Lareb Intensive Monitoring (LIM)] to a reference diabetes population. LIM patients were more often men, were generally younger and healthier (meaning that the sample included a higher percentage of *de novo* treated patients), had shorter diabetes duration, and used less co-medication than the reference population. The authors concluded that these differences might lead to underestimations of ADRs. In another study, Härmark et al. (2013c, 2014) investigated the reasons for patient non-response to invitations to participate in active monitoring systems. Among first-time users of antidiabetics, they found that patients who agreed to participate were on average 4.5 years younger than non-respondents and used less co-medication. No gender differences were found. The main reason for not participating was lack of information at the pharmacy. However, given the relatively high response rate to the posted questionnaire, the authors propose that patients are more willing to participate when they are informed and invited in the pharmacy.

In conclusion, although patients who participate in web-based intensive monitoring of drugs show little difference from reference populations, studies have demonstrated its usefulness for monitoring ADRs. It is therefore a complementary method to spontaneous reporting. It is also more proactive, in that it allows obtaining information on the evolution of ADRs over time and calculating the incidence for particular ADRs. The drawbacks are that it includes a limited number of patients and requires more resources to manage.

In addition to using web-based systems to gather spontaneous ADR reports and intensive monitoring systems for certain patients, it has been proposed that comments posted on Internet forums about the pros and cons of drugs can be analyzed.

### Analysis of Online Forum Comments

Schröder et al. (2007) consider online forums a suitable source of observational information to complement data from randomized clinical trials. They analysed drug-related problems in outpatient treatment of Parkinson’s disease that were posted anonymously on online forums. From the postings, they identified a number of drug-related problems that were otherwise largely invisible. These were mainly associated with qualitative treatment aspects such as medication handling, dosage, and individual ADRs. They also identified a number of differences between the forum postings and clinical study data. The online forums focused more on ADRs that the patients found particularly worrisome. For example, they reported more ADRs that affected the skin, including scarring, compared to expectations based on clinical trials. On the other hand, patients did not recognize ADRs that affected the metabolic system, for example, without a physician’s diagnosis, and these were not mentioned on the forum.

White et al. (2013) also hypothesized that Internet users might provide early clues about ADRs. Based on the premise that people would search for information about their conditions and medications online, they analyzed the Web search log data (Google, Bing, and Yahoo!) of 80 million consenting users over 18 months. The results showed that search log mining can contribute to drug safety surveillance. In a subsequent study, White et al. (2014) confirmed that the performance of ADR detection via search logs is comparable and complementary to detection using the adverse event reporting system (AERS) of the USA’s Food and Drug Administration (FDA). They showed that when these two types of data are combined, ADR detection accuracy can be improved by 19% over the use of each data source independently.

Similarly, Abou Taam et al. (2014) suggest that analyses of data from Internet sites can provide useful complementary information. They analyzed data from three French websites that featured discussion lists or forums on health issues. Their objective was to explore patients’ perceptions of the risks associated with benflurorex before, during, and after it was withdrawn from the market. Before the drug was withdrawn, most posted comments concerned treatment effectiveness. Once the risk for valvulopathy was broadcast in the media, most of the postings contained warnings or ADR reports. Afterwards, most of the comments expressed anger about the healthcare system, or anxiety about undesirable cardiovascular effects. Using a similar approach, Chee et al. (2009) used personal health messages from online communities to show that trends in people’s positive and negative feelings about particular drugs can be tracked over time. Changes in feelings corresponded to FDA announcements (e.g., market withdrawal of a drug) and other forms of publicity. For example, after Tysabri was introduced [to treat multiple sclerosis (MS)], feelings were initially positive, then became increasingly negative during a recall period, and subsequently returned to positive (albeit slightly less positive than originally) after it was reintroduced. The authors propose that this type of analysis can ultimately lead to the development of population health measurement tools to predict treatment outcomes. Thus, overall negative feelings about a particular drug or trend changes may indicate dissatisfaction, warranting further investigation into the causes of dissatisfaction, including ADRs.

Wu et al. (2013) found that online discussions can be monitored to detect previously unrecognised ADRs. They developed new methods for building an integrated knowledge base for ADR-related information and for monitoring online discussions about drugs and found that both methods can identify ADRs for four recently recalled drugs. Moreover, online discussions made it easier to detect ADRs much earlier than official announcements.

Pages et al. (2014) showed that data from website forums are considered relevant and worth sharing, and can be used to complement more conventional pharmacovigilance methods to detect AEs related to oral antineoplastic (OAN) drugs. However, some differences were found between AEs for OAN drugs reported on online forums and those recorded in the French pharmacovigilance database (FPVD): more musculoskeletal disorder reports were posted on patient websites, along with fewer skin disorders, and AEs reported on patient websites were less serious.

Several studies have addressed the contribution of discussions on social networking sites such as Facebook and Twitter.

Freifeld et al. (2014) evaluated the content of Twitter posts concerning possible AEs. Of 6.9 million Twitter posts, 4,401 possible AEs (Proto-AEs) were identified out of 60,000 examined. Overall, the Proto-AEs reported on Twitter showed similar distribution profiles to data from the traditional pharmacovigilance system (the above-mentioned FAERS). The authors concluded that the patients who reported on Twitter showed a range of sophistication when describing their experience, and that the contributory role of this type of information has yet to be clearly established. After assessing the potential of a using a Facebook group to identify potential ADRs, Knezevic et al. (2011) feel that such groups could be used to stimulate spontaneous ADR reporting.

The above-presented studies demonstrate that comments posted on online forums can be analyzed to gather information on potential ADRs. Notably, they can improve the understanding of patients’ experiences and concerns. In addition, the two studies by White et al. (2013, 2014) show that search log analysis produces valuable data. For instance, according to Noren (2014), many people who would hesitate to share adverse experiences in public might use Internet search engines to look for relevant information, and might agree to share this type of data.

Web-based data analysis may be considered a complementary information source to spontaneous reports and intensive monitoring of particular medications.

In addition to these three monitoring methods, all of which rely on the Internet to gather information, other pharmacovigilance methods that use other ehealth technologies have been explored, and mobile phones in particular.

### Use of Mobile Ehealth Systems (Cell Phones, Smartphones, and Tablets) to Monitor ADRs

In Europe, some enterprises are developing user-friendly apps that enable everyone to use a smartphone or tablet to make spontaneous ADR reports. However, these mobile ehealth systems are more often found in developing countries.

According to Gaur (2011), cellular technology provides an inexpensive means of monitoring ADRs in the developing world. The lack of resources in these parts makes it difficult to carry out large-scale pharmacovigilance programs. At the same time, cellular phones are in widespread use in such countries.

For example, Adedeji et al. (2011) showed that telephones can be used to obtain information on ADRs from individuals who purchased antimalarials. Participants were actively monitored by calls to their mobile phones throughout a 28-days monitoring period. The response rate was 57% in the first 24 h after purchase, dropping to 33% by day 4. The authors did not find any increase in known ADRs during the monitoring period. They suggest that mobile phones provide a practical means to report ADRs related to antimalarials, and could be the method of choice in Africa, where cell phones and cell phone coverage are currently driving innovative agricultural and trade practices.

Similarly, Baron et al. (2013) showed that mobile phone-based tools, and particularly short message service (SMS) text messages, can be used to gather information on ADRs following vaccination. In spring 2012 they recruited 184 participants from an international vaccination center. Two days after vaccination, participants received an automatically generated SMS text asking whether any AEs had occurred. Of those who agreed to participate, 28.3% did not reply, 54.9% sent an immediate SMS reply, and 16.8% sent a reply after more prompting. The authors concluded that an SMS-based system can be useful for gathering patients’ notifications, particularly in an urban setting. In Saudi Arabia, Aljadhey et al. (2012) corroborated the feasibility of gathering information on potential ADRs associated with the H1N1 vaccine in children via mobile phone contact with child caregivers.

Curioso et al. (2005) previously showed in a pilot project that cell phones could be used for real-time collection of ADRs in urban and rural Peru. Their objective was to develop an interactive computer-based system using cell phones to collect data on ADRs related to metronidazole use (to treat bacterial vaginosis) in prostitutes. However, in their study, public health workers reported the ADRs, and not prostitutes themselves. The ADRs were reported as codes via cell phones and stored in a secure online database. Data were immediately accessible worldwide, and could be analyzed and transmitted by email and SMS to team leaders to provide real-time alerts.

In sum, mobile phones have been demonstrated useful for gathering information on drug safety, especially in developing countries. Such ehealth systems show great promise for developing countries, where access to cell phones is more readily available compared to Internet-enabled computers. Nevertheless, studies have shown that responses tend to decrease over time, which hinders long-term follow-up.

## Discussion

It is increasingly recognized that patients can play a major role in identifying, describing, and preventing drug-related harm. Patients can contribute in several ways: they can submit spontaneous reports to pharmacovigilance agencies, participate in postmarketing monitoring programs and studies, and posts warnings and comments on online forums. In addition, in order to get patients involved, ehealth technologies are gaining in use, including websites, online forums, cell phones, apps, and surely more to come.

In recent years, patients have had access to more and more ways to make online reports of ADRs. Moreover, many studies have demonstrated that patient reports have provided useful information that complements reports by HCPs, and that can help identify potential new ADRs. Therefore, patient participation should improve pharmacovigilance. The data they provide is useful for combining with the data gathered in postmarketing monitoring studies, including intensive monitoring with patient follow-up over time as well as more specific studies. In addition, studies are making greater use of ehealth technologies. For instance, questionnaires can be emailed to patients, or patients can be contacted on their cell phones. Finally, patients’ online forum postings about the risks and benefits of medications can round out the picture. Each of these methods has its pros and cons (see the **Table 1** below), but taken together, they provide as complete a representation of ADRs as possible at this time.

**Table 1:** Pros and cons of the various methods for gathering pharmacovigilance data.

Pros	Cons
**Web-based spontaneous reporting of adverse drug reactions (ADRs).**	
–Faster and easier via the Internet than written reports.	–Targets individuals with ready Internet access, and is consequently unrepresentative of populations.
–More accessible for individuals with Internet access.	–Requires investment on the part of patients (taking the time to report ADRs).
–Requires fewer resources to manage data collection.	–Accuracy and reliability of reports are difficult to verify.
	–Patient identity is difficult to verify. Are they actually patients?
**Intensive drug monitoring studies.**	
–Other aspects can be gathered at the same time: medication use, dosage taken, compliance, perceived effectiveness.	–Targets individuals with ready Internet access.
–Allows gathering longitudinal data.	–Participating patients are not necessarily representative of the reference population.
	–Requires more resources to gather data (patient recruitment, emailing, etc.).
	–Studies generally include a limited number of patients, and are consequently not accessible to large populations.
**Analysis of online forum postings.**	
–Individuals who post comments are not required to follow a particular procedure or approach.	–Not everyone likes to post their comments on online forums. Only individuals who are willing to their experiences online will participate. Therefore, comments are unrepresentative of the population.
–The data are already shared on the Internet. Consequently, patients are not required to do anything further.	–Targets individuals with ready Internet access.
–The data are already online, and therefore do not need to be gathered.	–Does not allow follow-up over time.
–Allows rapid feedback on new-to-market drugs.	–Risk of excluding individuals such as the elderly or illiterate.
	–Impossible to verify the accuracy or reliability of comments.
	–The analysis of forum comments is complex and time-consuming.
**Use of mobile phone systems to monitor drug effects.**	
–Allows reaching a wide population worldwide, including developing countries.	–Excludes individuals without access to a cell phone, smartphone, or tablet.
–Allows reaching individuals immediately at any location.	–Risks excluding the elderly.
	–Requires more resources to gather data (patient recruitment, SMS messaging, etc.).
	–Studies generally include a limited number of patients, and are consequently not accessible to large populations.


Despite the promise of these innovative pharmacovigilance methods, a few issues remain unaddressed (see **Table 2**).

**Table 2:** State of the art, avenue for further development and issues that remain unresolved.

**State of the art.**
Recent developments in pharmacovigilance.
–Patient participation.
–The ability to make spontaneous ADR reports online.
–Web-based intensive drug monitoring studies.
–Analysis of online forum postings.
–The use of mobile ehealth systems to monitor ADRs.
**Avenues for further development.**
Future challenges.
–Raise patients’ awareness that they can report ADRs themselves.
–Raise awareness among patients and healthcare professionals of the importance of reporting ADRs so as to increase the number of reports.
–Standardise report questionnaires across countries to facilitate comparison and coding of data stored in databases.
–Rally all sections of the population to participate: the elderly, lower socioeconomic status groups, and the less educated.
–Explore strategies to generate online message sharing via health-specific social sites such as PatientsLikeMe, MedHelp, Inspire, CureTogether, etc.
–Make better use of databases generated by various methods (data mining issues).
**Issues that remain unresolved.**
Risks and liabilities
–Under-reporting of ADRs.
–Inherent differences between patient and healthcare professional reports.
–The population selection bias involved in many data collection methods.
–Overlooking the fact that the different ways to gather information on ADRs are complementary.
–The complexity of analyzing data gathered from online discussions and search engines.
–Data security and confidentiality issues for databases.


Generally, studies that analyzed spontaneous ADR reports considered all reports made by patients and HCPs, including paper, phone, and online reports. Therefore, it is unknown whether the differences observed between patient and HCP reports would vary (or not) according to the reporting mode. It is also unknown whether the ability to make online reports has increased the number of ADR reports, and whether patients as well as HCPs tend to use online reports more than paper or phone reports. With respect to cell phone apps that allow spontaneous reports anywhere, anytime, no study to date has specifically assessed the usefulness and added benefits of this technology.

Although online reports have certain advantages, notably more rapid data processing, they also have some drawbacks. It is more difficult to verify whether the ADR reporter is actually a patient, that the patient is taking the medication properly, and that the ADR has been reported just one time. Some people, intentionally or not, may report ADRs by mistake, or they may be mistaken in their perception of the ADR. Furthermore, patient-reported ADRs are usually not validated by a physician’s opinion. Moreover, some ADRs are undetected in medical exams, and may be undetected by the patient as well. In these cases, patient-reported information would be questionable. Yet decision makers need reliable information. Despite all these caveats, however, patient-generated data can be useful, especially for developing hypotheses about potential side-effects.

There is also the possibility of bias related to certain characteristics of patients who participate in pharmacovigilance, especially when ehealth tools are involved. For instance, data are gathered less often from older patients and those with lower socioeconomic status and less education. And, because pharmacovigilance centers are tending to use electronic reports, there is a risk of excluding individuals without Internet access or who are inept at using it. Some web-based postmarketing monitoring studies have demonstrated that participating patients are younger and have fewer pathologies. Similarly, the profiles of those who post comments on online forums should be taken into consideration. For example, we might suppose that fewer older individuals would participate in such forums. Adams (2013) examined comments posted on a Dutch website about patients’ experiences with medications. One potential drawback to the use of this type of information for pharmacovigilance is user representativeness. For instance, users who contribute to the site need to have a certain level of computer expertise, knowledge about health, and the ability to describe their experiences so that it is useful for others. The author also notes that more medications are consumed by certain demographic groups such as the elderly, people of low socioeconomic status, and patients with certain chronic conditions. As evidenced by the characteristics of site users, these are generally not the people who share their experiences online. Therefore, the comments cannot be considered entirely representative of the very groups for whom pharmacovigilance is the most important. Similarly, the Institute for Healthcare Informatics (IMS) (Gauntlett et al., 2013) found that recently developed apps are not well suited for patients with multiple chronic conditions, who are usually over 65 years old. Yet these are exactly the patients who are liable to be the biggest healthcare consumers. What is more, this group contains the smallest percentage of cell phone users, at only 18% of all users in the US compared to 54% of 45- to 54-year-olds.

For studies that address cell phone monitoring in developing countries, it would be equally important to analyze the profiles of people who use a cell phone and who are able and willing to respond to surveys. This could help determine whether or not the probability of participating in such studies is associated with age, socioeconomic status, and education level.

## Conclusion

This article confirms the value of involving patients themselves in pharmacovigilance as well as the usefulness of ehealth tools. Ehealth solutions facilitate data gathering from patients and HCPs alike. As such, they make a significant contribution to the detection of AEs and ADRs. In addition, worldwide collection of AEs using smart phones might become the leading technique in Low and Middle Income Countries where broad mobile phone service can be managed cheaper than Internet communication.

In future, initiatives are needed to better inform the public that they can make their own reports about ADRs, and the importance of doing so should be underscored. Strategies for engaging all sections of the population, including those who are less tech-adept, should be explored. Finally, the full potential of the databases generated by diverse ADR gathering methods should be leveraged.

## Author Contributions

JB, LD, and MD did substantial contributions to conception and design and analysis and interpretation of data. JB and MD participated in drafting the article. JB, LD, PO, and MD revisited the article critically for important intellectual content.

## Conflict of Interest Statement

The authors declare that the research was conducted in the absence of any commercial or financial relationships that could be construed as a potential conflict of interest.
